# The Long-term Effect of Different Exercise Intensities on High-Density Lipoprotein Cholesterol in Older Men and Women Using the Per Protocol Approach: The Generation 100 Study

**DOI:** 10.1016/j.mayocpiqo.2021.07.002

**Published:** 2021-09-16

**Authors:** Ida Berglund, Elisabeth Kleivhaug Vesterbekkmo, Kjetil Retterstøl, Sigmund A. Anderssen, Maria A. Fiatarone Singh, Jørn W. Helge, Stian Lydersen, Ulrik Wisløff, Dorthe Stensvold

**Affiliations:** a Department of Circulation and Medical Imaging, Faculty of Medicine and Health Sciences, Norwegian University of Science and Technology, Trondheim, Norway; b Regional Center for Child and Youth Mental Health and Child Welfare, Department of Mental Health, Faculty of Medicine and Health Sciences, Norwegian University of Science and Technology, Trondheim, Norway; c Department of Cardiology, St. Olavs Hospital, Trondheim University Hospital, Trondheim, Norway; d Department of Nutrition, Institute for Basic Medical Sciences, University of Oslo, Oslo, Norway; e The Lipid Clinic, Oslo University Hospital Rikshospitalet, Oslo, Norway; f Department of Sports Medicine, Norwegian School of Sport Sciences, Oslo, Norway; g Sydney School of Health Sciences and Sydney Medical School, The Faculty of Medicine and Health, The University of Sydney, Lidcombe, Australia; h Hinda and Arthur Marcus Institute for Aging Research, Hebrew SeniorLife, Boston, MA; i Xlab, Center for Healthy Aging, Department of Biomedical Sciences, Faculty of Health and Medical Sciences, University of Copenhagen, Copenhagen, Denmark; j School of Human Movement and Nutrition Science, University of Queensland, Brisbane, QLD, Australia

**Keywords:** CON, control, CVD, cardiovascular disease, HDL-C, high-density lipoprotein cholesterol, HIIT, high-intensity interval training, HRpeak, peak heart rate, LDL-C, low-density lipoprotein cholesterol, LMA, lipid-modifying agent, MICT, moderate-intensity continuous training, Vo_2_peak, peak oxygen uptake

## Abstract

**Objective:**

To examine whether 5 years of high-intensity interval training (HIIT) increases high-density lipoprotein cholesterol (HDL-C) concentration more than moderate-intensity continuous training (MICT) and control (CON) in older men and women.

**Methods:**

A total of 1567 older adults (790 [50.4%] women) were randomized (2:1:1) to either CON (n=780; asked to follow the national recommendations for physical activity) or 2 weekly sessions of HIIT (10-minute warm-up followed by 4×4-minute intervals at ∼90% of peak heart rate) or MICT (50 minutes of continuous work at ∼70% of peak heart rate). Serum HDL-C concentration was measured by standard procedures at baseline and at 1 year, 3 years, and 5 years. The study took place between August 21, 2012, and June 31, 2018. Linear mixed models were used to determine between-group differences during 5 years using the per protocol approach.

**Results:**

Men in HIIT had a smaller reduction in HDL-C (−1.2%) than men in CON (−6.9%) and MICT (−7.8%) after 5 years (*P*=.01 and *P*=.03 for CON vs HIIT and MICT vs HIIT, respectively). No effect of exercise intensity on HDL-C was seen in women. Changes in peak oxygen uptake were associated with changes in HDL-C in both men and women, whereas changes in body weight and fat mass were not.

**Conclusion:**

In men, HIIT seems to be the best strategy to prevent a decline in HDL-C during a 5-year period. No effect of exercise intensity was seen for older women.

**Trial registration:**

ClinicalTrials.gov identifier: NCT01666340.

Aging is characterized by functional and physiologic changes^1^^,^^2^ that increase the risk for development of chronic diseases, such as cardiovascular disease (CVD).^3^ Cardiovascular disease is the leading cause of death in older adults, and has globally been the world’s biggest killer the last 15 years.^4^ Dyslipidemia, defined as abnormalities in the standard lipid profile, is one of the major risk factors for CVD,^5^ and lipid-modifying agents (LMAs), such as statins, are the most commonly prescribed medication in older adults, acting as both primary and secondary prevention.^6^ The pharmacologic treatments effectively decrease low-density lipoprotein cholesterol (LDL-C) concentration; however, the effect on high-density lipoprotein cholesterol (HDL-C) concentration is controversial.^7^

Low levels of HDL-C are associated with cardiovascular events and mortality^8^ even when LDL-C levels are low,^5^ and improvements in HDL-C and triglyceride levels, in the absence of changes in LDL-C, reduce cardiovascular events.^9^ HDL-C is important in removal of cholesterol from peripheral cells to the liver (reverse cholesterol transport), is anti-inflammatory, and inhibits lipid oxidation.^1^ Increasing age slows or reverses several of these processes, leading to disordered lipid metabolism and systemic inflammation that further increases the risk of CVD.^1^ One potential approach for increasing HDL-C concentration is aerobic exercise,^10^ and high-intensity interval training (HIIT) has been found to be more effective at improving HDL-C concentration than moderate-intensity continuous training (MICT) in younger adults.^11^ However, the effect of exercise on HDL-C in older adults is unclear.^12^ It has been suggested that older adults need a longer time to adapt positively to exercise with respect to HDL-C.^13^ In addition, few studies have examined the effect of identical exercise programs in men and women on HDL-C, and more knowledge is needed to determine whether the optimal training program for older men and women differs with respect to HDL-C adaptation.^12^^,^^14^

The primary aim of this study was to test the hypothesis that 5 years of HIIT increases HDL-C concentration more than MICT and control (CON). We also examined a secondary hypothesis that changes in HDL-C are associated with changes in peak oxygen uptake (Vo_2_peak), body weight, and fat mass after 5 years of exercise. Only participants who adhered to the prescribed exercise intervention were included in this study (ie, per protocol approach).

## Methods

### Study Population and Design

**Figure 1:**
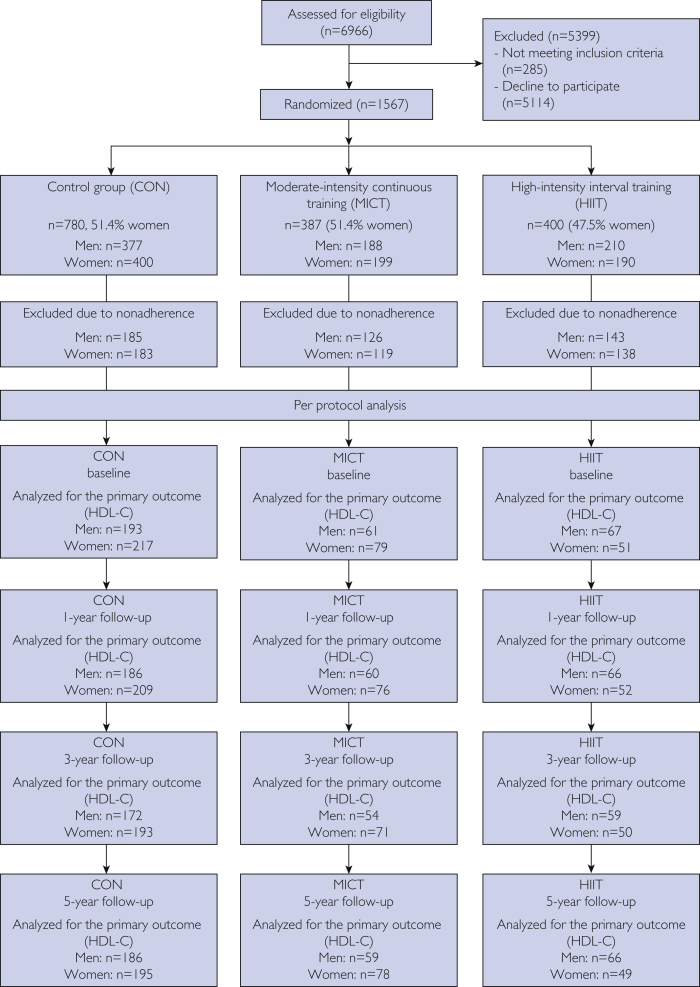
Flowchart. Adherence to control (CON), moderate-intensity continuous training (MICT), and high-intensity interval training (HIIT) represents the proportion of men and women who adhered to the prescribed exercise program during 5 years and the proportion of men and women who were analyzed for the primary outcome at each follow-up time. HDL-C, high-density lipoprotein cholesterol.

In 2012, all men and women between the ages of 70 and 77 years with a permanent address in the municipality of Trondheim were invited to participate in a randomized controlled trial, the Generation 100 study.^15^ Participants were excluded if they had illnesses or disabilities, including cancers and dementia, that prevented exercise or hindered completion of the study. Furthermore, participants with chronic communicable infectious diseases and symptomatic valvular heart disease, hypertrophic cardiomyopathy, unstable angina, primary pulmonary or uncontrolled hypertension, heart failure, or severe arrhythmias were excluded.^15^ Recruitment, intervention program, and laboratory procedures of the Generation 100 study have been described in detail elsewhere.^15^ In short, a total of 777 men and 790 women were included in the study and were examined by questionnaires, clinical examinations, blood sampling, and physical tests at baseline and after 1 year and 3 years. Participants were randomized (2:1:1), stratified by sex and cohabiting status, after the health examination at baseline to CON (n=780), MICT (n=387), or HIIT (n=400) for 5 years. The randomization process was performed by the unit for Applied Clinical Research at the Norwegian University of Science and Technology. Personnel were blinded to intervention group during testing. For this particular study, we used a per protocol approach, and participants were included if they had 50% or more adherence to the prescribed exercise intervention during 5 years. After 5 years, 673 participants (350 [52%] women) adhered to the prescribed exercise program or to the national recommendations for physical activity and were included in the study. This included 412 participants (218 women) in CON, 142 participants (80 women) in MICT, and 119 (52 women) in HIIT. The study was approved by the regional ethics committee before study start (REK2012/381 B), and the study was registered in ClinicalTrials.gov in August 2012. The participants signed an informed consent form that covers the aim and objectives of the study (REK2018/2138-1). A flowchart of the study is presented in Figure 1.

### Clinical and Biochemical Testing

Participants were asked to be fasting and to avoid tobacco, alcohol, and caffeine for 12 hours and exercise for 24 hours before examination. Blood sampling was standardized and obtained in a sitting position from the left arm vein. Serum HDL-C concentrations were measured immediately by standard procedures at St. Olavs Hospital, Trondheim, Norway. The laboratory at St. Olavs Hospital is under a laboratory quality management system program, and quality assurances were thereby performed frequently. Weight and fat percentage were measured by bioelectrical impedance (Inbody 720, BIOSPACE). Participants with a pacemaker had weight measured on a regular scale. Fat mass was calculated by multiplying fat percentage and body weight divided by 100.

Testing of Vo_2_peak was performed on a treadmill (n=656) or a stationary bike (n=13) at the NeXt Move Core Facilities, St. Olavs Hospital, Norway. Stationary bikes were used only for participants who were unable to walk on the treadmill (eg, unstable or acute injury). We mainly used Cortex MetaMax II (n =641) and used Oxycon Pro (Erich Jaeger; n=28) as a backup for days with technical difficulties. The same system was used for each participant at all follow-ups. The testing and calibration procedures have been described previously.^15^ In short, a facemask (Hans Rudolph) connected to the gas analyzer was attached to the participants before initiation of the test. After 10-minute warm-up at moderate intensity, an individualized protocol was used whereby the load was increased each 1.5 minutes by 1 km/h or 2% inclination. The test was terminated voluntarily or with a plateau in oxygen uptake (Vo_2_) with increased load (ie, no more than 2 mL·min^−1^·kg^−1^ between two 30-second segments). Peak oxygen uptake was the average of the 3 highest values during the test. Participants with previous heart diseases were tested under electrocardiographic monitoring, and the American College of Cardiology/American Heart Association guidelines for exercise testing of patients with known CVD were observed.^16^ Peak heart rate (HRpeak) was determined as the highest measured heart rate during the Vo_2_peak test. Peak heart rates measured during a Vo_2_peak test have had good agreement with the maximum heart rate measured in a test designed to measure maximum heart rate in younger adults.^17^

### Intervention

The HIIT group warmed up for 10 minutes at moderate intensity at approximately 70% of HRpeak. Immediately after warm-up, the participants continued with four 4-minute bouts performed at approximately 90% of HRpeak, interspersed with 3 minutes of active recovery at approximately 70% HRpeak. The session ended with a 5-minute cool-down at approximately 70% HRpeak. The MICT group performed a continuous workout at an intensity of approximately 70% of HRpeak for 50 minutes. Both exercise groups were offered supervised exercise sessions twice a week for 5 years. Participants were free to choose the preferred exercise mode (ie, walking, running, cycling), but they met for 1 mandatory supervised spinning session every sixth week. Heart rate monitors and the Borg scale (6-20)^18^ were used to promote recommended exercise intensity. The control group was encouraged to follow the national recommendations for physical activity in 2012, which included 30 minutes of physical activity on most days of the week.^19^ All groups received verbal and written information at inclusion and at each follow-up time point.

### Self-Reported Questionnaires

Adherence to the exercise program was determined from questionnaires previously described,^15^ including exercise frequency—How often do you exercise? never or less than once per week [0], once a week [1], 2 or 3 times a week [2.5], nearly every day [5]; duration—For how long do you exercise each time? less than 15 minutes [7.5], 15 to 30 minutes [22.5], 30 to 60 minutes [45], more than 60 minutes [60]; and intensity using the Borg scale—On a scale of 6 to 20, how hard do you exercise?^20^

Minutes per week were calculated by multiplying frequency and duration (averages denoted in brackets).

As previously described,^21^ the minimum requirements for adherence to HIIT were at least 30 minutes of weekly exercise at 15 or higher on the Borg scale, and the requirements for adherence to MICT were at least 30 minutes of weekly exercise at 11 to 14 on the Borg scale. Adherence of the CON group was determined as 30 minutes or more of weekly physical activity at any intensity. This included the criteria for inclusion in the study and had to be followed for all 5 years.

Information on smoking status and alcohol consumption was determined from questionnaires previously described.^15^ Smoking status (Do you smoke? No, I have never smoked; No, I quit smoking; Yes, cigarettes occasionally [parties/vacation, not daily]; Yes, cigars/cigarillos/pipe occasionally; Yes, cigarettes daily; Yes, cigars/cigarillos/pipe daily) was dichotomized to former smoker and current smoker. Alcohol consumption (How many glasses of beer, wine, or spirits do you usually drink in the course of 2 weeks? and How often do you drink 5 glasses or more of beer, wine, or spirits in one sitting? never; monthly; weekly; daily) was described as average units of alcohol per week and dichotomized (yes/no) to binge drinker, defined as 5 units or more of alcohol weekly. Use of fish oils, omega-3 supplements, and vitamin supplements (How often do you usually take fish oil/omega-3/vitamin supplements? never/rarely; 1 to 3 times/month; 1 to 3 times/week; 4 to 6 times/week; 1 daily; 2 times daily; 3 times daily; 4 or more times per day) at baseline was categorized to yes/no.

### Cardiovascular Disease and Lipid-Modifying Agents

Information on use of prescribed medications was obtained from the participants’ medical prescription registry. Medication affecting lipid metabolism was defined as LMAs (Anatomical Therapeutic Chemical classification code C10). In addition, history of CVD at inclusion into the study was obtained from the participants’ archive at St. Olavs Hospital.

### Statistics

Descriptive continuous data are presented as mean and standard deviation and categorical variables as proportion and number of participants. We used linear mixed models with the outcome HDL-C and secondary outcomes (body weight and Vo_2_peak), 1 at a time, as dependent variables. The individual was included as a random effect. We included time, intervention group (CON vs MICT vs HIIT and MICT vs HIIT), and sex and their 2-way (time × group, time × sex, and group × sex) and 3-way (time × group × sex) interactions as categorical covariates. All analyses were adjusted for age and cohabiting status. Because of non-normally distributed residuals, we used bootstrapping with the bias corrected and accelerated method, using 2000 bootstrap samples. The analyses were carried out by the per protocol approach and included participants in MICT and HIIT with 50% adherence to the prescribed exercise program and participants in CON who adhered to the national recommendations for physical activity for the entire duration of the study. We performed subanalyses whereby participants with CVD or using LMAs at inclusion were excluded. Forward linear regression was used to examine the association between changes in Vo_2_peak (ΔVo_2_peak), fat mass (ΔFM), and body weight (Δweight) and changes in HDL-C (ΔHDL-C) in older men and women. Delta (Δ) values were calculated by subtracting baseline values from the year 5 values. The dependent variable was ΔHDL-C, and ΔVo_2_peak, ΔFM, and Δweight were used as independent variables, and we adjusted for smoking status (yes/no).^22^ A separate model was run for men and women. As some participants changed between treadmill and ergometer bike when performing the Vo_2_peak test, these were excluded from the analysis (n=3) to ensure that changes in Vo_2_peak were not due to changed modality. A 2-sided *P* value of less than .05 was considered statistically significant. Because of multiple comparisons, *P* values between 1% and 5% must be interpreted with caution. All statistical analyses were performed using SPSS version 25 (IBM). Sample size for the main study was calculated on the basis of mortality.^15^

## Results

### Baseline Characteristics

Baseline characteristics of the participants are presented in Table 1. The average age (mean [standard deviation]) was 72.5 (1.9) years for men and 72.6 (2.1) years for women. Women had on average higher HDL-C levels than men (1.94 [0.51] vs 1.58 [0.42] mmol/L, respectively) and lower Vo_2_peak than men (27.41 [4.98] vs 33.13 [6.45] mL·min^−1^·kg^−1^, respectively) at inclusion.

**Table 1:** Baseline Characteristics of Men and Women in Each Intervention Group^a^^,^^b^

	CON		MICT		HIIT	
	Men (n=194)	Women (n=218)	Men (n=62)	Women (n=80)	Men (n=67)	Women (n=52)
Age (y)	72.5 (1.9)	72.6 (2.1)	72.7 (2.1)	72.6 (2.0)	72.3 (1.9)	72.8 (2.1)
HDL-C (mmol/L)	1.59 (0.43)	1.95 (0.51)	1.53 (0.40)	1.93 (0.49)	1.61 (0.42)	1.90 (0.52)
LDL-C (mmol/L)	3.21 (0.93)	3.68 (0.99)	3.31 (1.05)	3.53 (0.97)	3.19 (0.94)	3.43 (1.03)
TC (mmol/L)	5.33 (1.00)	6.13 (1.02)	5.36 (1.16)	5.95 (1.01)	5.29 (1.00)	5.85 (1.13)
TG (mmol/L)	1.16 (0.57)	1.11 (0.56)	1.16 (0.65)	1.08 (0.55)	1.08 (0.49)	1.15 (0.61)
Weight (kg)	81.5 (9.8)	66.9 (10.2)	81.8 (12.0)	67.5 (9.8)	81.5 (11.5)	66.4 (10.0)
Vo_2_peak (mL·min^−1^·kg^−1^)	32.95 (6.55)	27.45 (4.90)	32.31 (5.57)	26.81 (5.14)	34.42 (6.82)	28.18 (5.03)
CVD	16.5 (32)	10.1 (22)	25.8 (16)	3.8 (3)	19.4 (13)	9.6 (5)
LMA	24.2 (47)	21.6 (47)	27.4 (17)	28.7 (23)	25.4 (17)	36.5 (19)
Smoking status Former Current	47.9 (93) 5.2 (10)	35.8 (78) 5.5 (12)	50.0 (31) 6.4 (4)	33.8 (27) 8.8 (7)	41.8 (28) 4.5 (3)	30.8 (16) 0.0 (0)
Binge drinker^c^	5.7 (11)	3.2 (7)	12.9 (8)	1.3 (1)	10.4 (7)	1.9 (1)
Alcohol (units/wk)	1.9 (1.0)	1.4 (0.9)	2.4 (0.9)	1.1 (0.9)	2.1 (1.3)	1.1 (0.9)
Fish oil^d^	45.4 (88)	31.2 (68)	35.5 (22)	27.5 (22)	37.3 (25)	36.5 (19)
Omega-3^d^	33.5 (65)	39.4 (86)	37.1 (23)	46.3 (37)	28.4 (19)	46.2 (24)
Vitamins^d^	34.5 (67)	49.5 (108)	32.3 (20)	48.8 (39)	34.3 (23)	40.4 (21)

a CON, control; CVD, cardiovascular disease; HDL-C, high-density lipoprotein cholesterol; HIIT, high-intensity interval training; LDL-C, low-density lipoprotein cholesterol; LMA, lipid-modifying agent; MICT, moderate-intensity continuous training; TC, total cholesterol; TG, triglyceride; Vo_2_peak, peak oxygen uptake.

b Continuous data are presented as mean (standard deviation), and categorical data are presented as percentage (number).

c Binge drinker: more than 5 units of alcohol in 1 sitting, weekly or more often.

d Supplements: proportion of participants taking supplements monthly or more often.

### Exercise Habits

Detailed exercise habits for men and women in each intervention group are presented in Table 2. After 5 years, there were no differences between the intervention groups in average exercise sessions per week and average duration of each exercise session. Both men and women in CON and MICT had an average intensity of 12 to 13 on the Borg scale of 6 to 20, and men and women in HIIT had an average intensity of 15 on the Borg scale of 6 to 20. Of men in CON, 57% and 37% fulfilled the criteria for MICT and HIIT, respectively. The corresponding numbers for women were 62% and 23%, respectively.

**Table 2:** Detailed Exercise Description of Participants Who Adhered to the Prescribed Exercise Program for 5 Years^a^^,^^b^

	CON		MICT		HIIT	
	Men	Women	Men	Women	Men	Women
Baseline						
Intensity^c^	13.8 (1.9)	13.1 (1.9)	12.6 (1.8)	12.7 (2.1)	14.4 (2.2)	13.6 (1.9)
Frequency (sessions/week)	2.7 (1.3)	2.9 (1.4)	3.1 (1.4)	2.9 (1.3)	2.7 (1.3)	2.8 (1.2)
Duration (min/session)	47.4 (11.5)	47.6 (10.3)	46.6 (11.1)	47.8 (10.2)	51.0 (8.3)	49.4 (9.0)
Year 1						
Intensity^c^	14.1 (1.8)	13.5 (1.6)	12.7 (1.2)	12.8 (0.9)	15.9 (1.3)	15.4 (1.0)
Frequency (sessions/week)	3.3 (1.3)	3.3 (1.3)	3.1 (1.3)	3.2 (1.2)	3.0 (1.1)	3.1 (1.1)
Duration (min/session)	48.4 (11.4)	47.3 (10.2)	46.5 (11.0)	47.3 (8.5)	51.0 (8.3)	48.0 (9.3)
Year 3						
Intensity^c^	14.1 (1.7)	13.6 (1.5)	12.8 (1.1)	13.0 (0.8)	15.9 (1.3)	15.3 (1.4)
Frequency (sessions/week)	3.3 (1.4)	3.3 (1.4)	3.1 (1.3)	3.1 (1.1)	3.0 (1.2)	3.0 (1.1)
Duration (min/session)	48.0 (11.3)	47.3 (9.9)	46.7 (11.6)	48.1 (9.5)	49.1 (10.1)	49.7 (10.0)
Year 5						
Intensity^c^	13.8 (1.8)	13.3 (1.7)	12.6 (1.2)	12.8 (1.1)	15.4 (1.2)	15.1 (1.8)
Frequency (sessions/week)	3.3 (1.4)	3.2 (1.5)	3.1 (1.3)	3.1 (1.2)	3.0 (1.2)	3.2 (1.3)
Duration (min/session)	47.2 (12.7)	46.2 (10.9)	46.8 (9.8)	48.8 (11.5)	49.9 (8.7)	48.0 (12.4)

a CON, control; HIIT, high-intensity interval training; MICT, moderate-intensity continuous training.

b Data are presented as mean (standard deviation).

c Exercise intensity is measured by the Borg scale (6-20) and is reported as average intensity per exercise session.

### Changes in HDL-C Over Time in Older Men and Women

**Figure 2:**
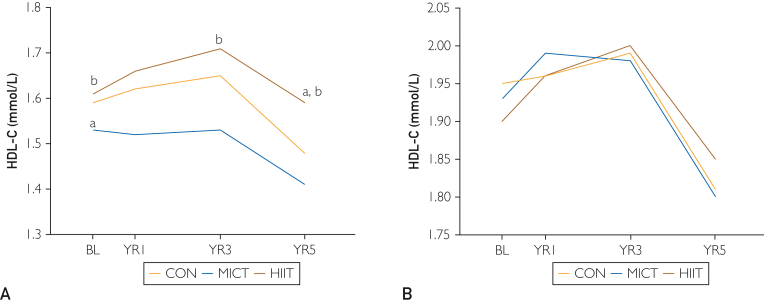
The levels of high-density lipoprotein cholesterol (HDL-C) in men (A) and women (B) from baseline to 5-year follow-up. A, *P*<.05 compared with control group (CON). B, *P*>.05 compared with moderate-intensity continuous training (MICT). BL, baseline; HIIT, high-intensity interval training.

The mean between-group differences and changes over time in HDL-C in men and women are presented in Table 3 and Figure 2. After 5 years, HDL-C concentration was significantly reduced in CON and MICT, for both men and women. Men reduced HDL-C concentration by 6.9% (*P*<.0001), 7.8% (*P*=.001), and 1.2% (*P*=.31) for CON, MICT, and HIIT, respectively. The reduction of HDL-C concentration was significantly lower for men in HIIT than for men in CON (*P*=.01) and MICT (*P*=.03) after 5 years. After 5 years, women reduced HDL-C concentration by −7.2%, −6.7% (both *P*<.0001), and −2.6% (*P*=.19) for CON, MICT, and HIIT, respectively; however, the difference between the groups did not reach statistical significance.

**Table 3:** The Exercise Effect on HDL-C in Men and Women^a^

		CON		MICT		HIIT		Group × Time MICT vs CON		Group × Time HIIT vs CON		Group × Time HIIT vs MICT	
Time^b^	Sex	No.	Mean (SD)	No.	Mean (SD)	No.	Mean (SD)	Estimate (95% CI)	*P* value	Estimate (95% CI)	*P* value	Estimate (95% CI)	*P* value
0	Men	193	1.59 (0.43)	61	1.53 (0.45)	67	1.61 (0.42)	−0.06 (−0.11, −0.02)	.01	0.02 (−0.03, 0.07)	.45	0.08 (0.02, 0.14)	.009
	Women	217	1.95 (0.51)	79	1.93 (0.49)	51	1.90 (0.52)	−0.02 (−0.07, 0.03)	.54	−0.06 (−0.15, 0.03)	.20	−0.04 (−0.14, 0.06)	.39
1	Men	186	1.62 (0.44)	60	1.52 (0.45)	66	1.66 (0.39)	−0.04 (−0.12, 0.04)	.37	0.002 (−0.07, 0.07)	.96	0.04 (−0.05, 0.12)	.42
	Women	209	1.96 (0.52)	76	1.99 (0.52)	52	1.96 (0.43)	0.04 (−0.05, 0.12)	.33	0.06 (−0.03, 0.16)	.25	0.02 (−0.10, 0.14)	.79
3	Men	172	1.65 (0.45)	54	1.53 (0.45)	59	1.71 (0.50)	−0.04 (−0.12, 0.04)	.30	0.06 (−0.01, 0.15)	.13	0.11 (0.01, 0.21)	.04
	Women	193	1.99 (0.52)	71	1.98 (0.48)	50	2.00 (0.44)	0.04 (−0.03, 0.12)	.27	0.07 (−0.02, 0.18)	.19	0.03 (−0.08, 0.16)	.65
5	Men	186	1.48 (0.38)	59	1.41 (0.33)	66	1.59 (0.41)	−0.004 (−0.07, 0.07)	.91	0.09 (0.02, 0.16)	.01	0.10 (0.01, 0.18)	.03
	Women	195	1.81 (0.47)	78	1.80 (0.42)	49	1.85 (0.39)	0.02 (−0.05, 0.09)	.54	0.08 (−0.02, 0.20)	.20	0.06 (−0.06, 0.19)	.39

a CON, control; HDL-C, high-density lipoprotein cholesterol; HIIT, high-intensity interval training; MICT, moderate-intensity continuous training.

b Years from inclusion.

We observed no changes in HDL-C in men or women after 1 year. However, men in CON and HIIT significantly improved HDL-C levels by 3.8% and 6.2%, respectively, after 3 years (*P*=.002 and *P*=.004, respectively), whereas no changes were seen in men in MICT at the same time point. Only the change in HIIT reached statistical significance from the change in MICT (*P*=.04) after 3 years. No changes in HDL-C levels were seen in women after 3 years.

The effect of time and intervention remained the same for HDL-C in the sensitivity analysis, in which participants with CVD or taking an LMA at inclusion were excluded (data not shown).

### Changes in Body Weight and Vo_2_peak Over Time in Older Men and Women

The between-group differences and changes in Vo_2_peak, body weight, and fat mass over time are presented in Table 4.

**Table 4:** The Exercise Effect on Vo_2_peak, Body Weight, and Fat Mass in Men and Women^a^

Time^b^	Sex	CON		MICT		HIIT		Group × Time MICT vs CON		Group × Time HIIT vs CON		Group × Time HIIT vs MICT	
		No.	Mean (SD)	No.	Mean (SD)	No.	Mean (SD)	Estimate (95% CI)	*P* value	Estimate (95% CI)	*P* value	Estimate (95% CI)	*P* value
Vo_2_peak (mL·min^−1^·kg^−1^)													
0	Men	193	32.95 (6.55)	61	32.31 (5.57)	67	34.42 (6.82)	−0.51 (−1.56, 0.46)	.26	1.67 (0.73, 2.57)	<.001	2.18 (1.11, 3.27)	<.001
	Women	214	27.45 (4.90)	79	26.81 (5.14)	51	28.18 (5.03)	−0.64 (−1.37, 0.09)	.07	0.83 (−0.25, 2.04)	.11	1.47 (0.20, 2.76)	.009
1	Men	185	34.30 (7.35)	59	34.09 (7.12)	67	37.55 (6.35)	0.31 (−1.05, 1.77)	.68	1.80 (0.49, 2.96)	.007	1.49 (−0.10, 3.03)	.08
	Women	209	29.28 (5.59)	77	28.57 (5.15)	51	30.99 (5.89)	−0.19 (−1.26, 0.82)	.68	0.99 (−0.47, 2.41)	.18	1.18 (−0.34, 2.70)	.15
3	Men	172	32.51 (7.78)	50	31.52 (6.52)	62	35.97 (7.41)	−0.45 (−1.81, 0.93)	.52	1.87 (0.46, 3.16)	.02	2.33 (0.65, 3.92)	.01
	Women	191	27.29 (5.43)	68	26.62 (5.61)	50	29.13 (5.43)	−0.05 (−1.07, 0.98)	.93	1.33 (−0.06, 2.63)	.06	1.38 (−0.17, 2.86)	.09
5	Men	175	31.31 (7.05)	52	30.34 (6.55)	62	33.52 (7.03)	−0.69 (−2.08, 0.71)	.35	0.85 (−0.55, 2.13)	.23	1.54 (−0.10, 3.03)	.08
	Women	183	26.39 (5.28)	70	26.17 (5.07)	49	28.20 (5.27)	0.37 (−0.55, 1.26)	.43	1.41 (0.09, 2.62)	.06	1.04 (−0.44, 2.41)	.18
Body weight (kg)													
0	Men	194	81.5 (9.8)	62	81.8 (12.0)	66	81.5 (11.5)	0.45 (−0.64, 1.39)	.24	−0.19 (−1.04, 0.51)	.61	−0.64 (−1.52, 0.09)	.18
	Women	218	66.9 (10.2)	80	67.5 (9.8)	52	66.4 (9.9)	0.45 (−0.24, 1.06)	.09	−0.46 (−1.37, 0.31)	.34	−0.91 (−1.92, −0.01)	.09
1	Men	192	80.9 (10.1)	62	80.5 (11.9)	67	79.9 (11.4)	−0.71 (−1.58, 0.18)	.12	−0.94 (−1.71, −0.09)	.03	−0.23 (−1.28, 0.91)	.66
	Women	216	66.3 (10.2)	79	66.1 (9.3)	51	64.5 (8.1)	−0.29 (−1.08, 0.49)	.46	−0.55 (−1.63, 0.53)	.28	−0.26 (−1.36, 0.87)	.67
3	Men	182	81.2 (10.0)	55	82.0 (12.5)	63	79.7 (9.8)	−0.02 (−1.09, 1.01)	.96	−0.93 (−1.83, −0.00)	.05	−0.91 (−2.03, 0.26)	.12
	Women	203	66.7 (10.3)	73	67.3 (9.9)	52	65.5 (8.5)	−0.32 (−1.11, 0.51)	.41	−0.91 (−2.15, 0.32)	.11	−0.59 (−1.88, 0.59)	.35
5	Men	187	80.7 (10.0)	59	81.4 (12.2)	66	80.2 (11.8)	0.03 (−1.03, 1.02)	.97	−0.55 (−1.57, 0.55)	.27	−0.58 (−1.76, 0.72)	.37
	Women	200	66.5 (10.4)	78	67.1 (10.3)	51	64.5 (7.9)	0.12 (−0.82, 1.17)	.80	−1.19 (−2.57, 0.18)	.07	−1.31 (−2.78, 0.13)	.07
Fat mass (kg)													
0	Men	192	20.1 (6.5)	62	20.6 (8.5)	66	20.5 (6. 7)	0.55 (−0.16, 1.32)	.11	0.34 (−0.26, 0.93)	.25	−0.21 (−0.97, 0.45)	.60
	Women	217	23.4 (7.8)	80	23.4 (7.4)	52	23.3 (7.0)	−0.03 (−0.60, 0.47)	.93	−0.17 (−0.91, 0.52)	.67	−0.14 (−1.04, 0.75)	.77
1	Men	189	20.1 (6.8)	62	20.0 (8.5)	65	19.7 (6.8)	−0.62 (−1.52, 0.27)	.17	−0.61 (−1.40, 0.15)	.12	0.01 (−0.97, 1.01)	.99
	Women	215	23.0 (7.6)	79	22.6 (6.9)	50	21.7 (5.9)	0.03 (−0.74, 0.83)	.93	−0.71 (−1.74, 0.31)	.12	−0.73 (−1.80, 0.36)	.16
3	Men	181	21.0 (6.9)	54	21.2 (8.7)	62	21.1 (6.9)	−0.49 (−1.41, 0.40)	.29	−0.20 (−1.02, 0.61)	.62	0.29 (−0.71, 1.33)	.59
	Women	200	23.7 (7.6)	73	23.8 (7.6)	52	22.7 (6.1)	−0.06 (−0.81, 0.69)	.87	−1.00 (−2.05, 0.05)	.05	−0.95 (−2.05, 0.20)	.01
5	Men	184	21.0 (6.7)	59	21.8 (9.0)	65	21.9 (7.5)	0.06 (−0.91, 1.07)	.90	0.24 (−0.66, 1.23)	.59	0.18 (−0.89, 1.36)	.74
	Women	198	23.6 (7.9)	78	24.0 (7. 8)	51	22.2 (5.8)	0.21 (−0.65, 1.04)	.63	−1.20 (−2.43, 0.09)	.05	−1.40 (−2.78, −0.06)	.04

a CON, control; HDL-C, high-density lipoprotein cholesterol; HIIT, high-intensity interval training; MICT, moderate-intensity continuous training; Vo_2_peak, peak oxygen uptake.

b Years from inclusion.

Body weight was significantly reduced for men in CON and HIIT (−1.0% and −1.6%; both *P*=.007) and for women in HIIT (−2.9%; *P*=.008) after 5 years. However, neither men nor women reached statistically significant between-group differences after 5 years. Only men in HIIT (−2.0%) had a larger reduction compared with men in CON (−0.7%) after 1 year (*P*=.03). No between-group differences were seen for women after 1 year.

After 5 years, women in HIIT reduced fat mass significantly more than women in CON and MICT (*P*=.05 and *P*=.04, respectively). Women in HIIT had a significantly larger reduction in fat mass compared with women in CON after 3 years. No between-group differences were observed in women after 1 year. We observed no between-group differences in men at any follow-up times.

After 5 years, Vo_2_peak was significantly reduced for men by −5.0%, −6.1% (both *P*<.0001), and −2.6% (*P*=.04) in CON, MICT, and HIIT, respectively. No between-group differences were observed after 5 years for Vo_2_peak in men. Women in CON and MICT significantly reduced Vo_2_peak by −3.9% (*P*<.0001) and −2.4% (*P*=.008), and no changes were observed for Vo_2_peak for women in HIIT after 5 years; however, the changes did not reach statistical significance between the groups. After 1 year, men in HIIT improved Vo_2_peak (9.1%; *P*<.0001) more than men in CON (4.1%; *P*=.001) (*P*=.007), and after 3 years, Vo_2_peak was improved more in men in HIIT (4.5%; *P*=.03) compared with men in CON (1.3%) and MICT (−2.4%; *P*=.02 and *P*=.011, respectively). No between-group differences were seen for women in Vo_2_peak during the study.

### Predictors of Changes in HDL-C

Regression analysis found that every 1 mL·min^−1^·kg^−1^ change in Vo_2_peak increased ΔHDL-C by 0.011 mmol/L (*P*=.001) in men. In women, 1 mL·min^−1^·kg^−1^ change in Vo_2_peak increased ΔHDL-C by 0.016 mmol/L (*P*<.001). Changes in HDL-C were significantly associated with ΔVo_2_peak in men (*F*_1,262_=10.835; *P*=.001; *R*^*2*^=0.036) and women (*F*_1,267_=12.845; *P*<.001; *R*^*2*^=0.042) after 5 years. Changes in body weight and fat mass were not associated with changes in HDL-C.

## Discussion

This study is the first to evaluate the effect of 5 years of different exercise intensities on HDL-C in a large population of older men and women using the per protocol approach. After 5 years, men had a smaller reduction in HDL-C in HIIT compared with CON and MICT. There were no differences in women. Changes in Vo_2_peak but not in body weight or fat mass were associated with changes in HDL-C in both men and women.

The effect of exercise on HDL-C in older adults is debated,^12^^,^^13^^,^^23^ and the variable results are often explained by the heterogeneity of exercise interventions.^12^^,^^24^ Studies including younger adults have reported that regular aerobic exercise improves HDL-C levels^25^ and that higher intensity is superior to moderate intensity for improving HDL-C concentration.^11^ In our study, we observed a smaller reduction in HDL-C concentration after 5 years in men in HIIT compared with men in MICT and CON. Exercise frequency and duration were similar between groups, and exercise intensity was the only exercise factor differentiating the 3 groups. It has been suggested that initial levels of HDL-C influence the training adaptation as more favorable levels at inclusion lead to smaller changes.^26^ Men in HIIT had initially higher HDL-C levels compared with the 2 other groups but did also have the largest improvements after 3 years and the only intervention that maintained HDL-C levels after 5 years. Thus, higher initial HDL-C levels did not affect the changes in older men. A longer intervention time has been proposed for older adults for exercise adaptation in lipids and lipoproteins.^13^^,^^27^ Interestingly, we observed an increase in HDL-C in men in HIIT and CON after 3 years, indicating that exercise has an effect on HDL-C in older men. However, only men in HIIT improved HDL-C concentration more than men in MICT. Surprisingly, the CON group had a relatively high level of physical activity throughout the study, and a relatively large percentage exercised with high intensity. The lack of significant differences between the HIIT and CON groups after 3 years could be due to the crossover between the interventions, whereby a relatively high proportion of the CON group exercised with high intensity. However, our data indicate that long-term exercise can improve HDL-C levels in older men. Our data are in line with those of King et al,^13^ who found no changes in HDL-C in middle-aged adults after 1 year but a small, significant increase after 2 years. Our result indicates that HIIT is more efficient than MICT in improving HDL-C levels after 3 years and more efficient than MICT and CON in maintaining HDL-C levels over time in older men. The same trend was seen in women; however, no changes were observed between the intervention groups as all groups were able to maintain their HDL-C levels.

It has been speculated that women have an attenuated response to exercise in regard to HDL-C compared with men.^28^ In our study, none of the interventions were superior in regard to HDL-C levels in women. Several previous studies have failed to find an exercise effect on HDL-C in older women.^29^, ^30^, ^31^ However, in a meta-analysis of younger, healthy women, HDL-C increased by 3% after exercise.^32^ The women in our study had initially high levels of HDL-C that previously have been found to influence the training response.^26^ Intensity is important to limit the reduction seen in HDL-C in men, but our results indicate that higher exercise intensity is not crucial for women. It could be that a higher exercise dose (ie, exercise frequency or duration) is more important for changes in HDL-C in older women, as seen in younger women.^33^

### Changes in Fitness and Body Composition

Current guidelines recommend weight loss to improve an abnormal lipid profile.^5^ Our data found that that reduction in body weight or reduction in fat mass is not necessary to improve HDL-C concentration in older men. However, the absolute change in our study was relatively small, and we cannot determine whether a larger reduction in body weight or fat mass could have led to improvements in HDL-C levels. It has previously been confirmed that changes in HDL-C are associated with changes in fat mass but not Vo_2_peak in younger adults.^22^ However, in the older adults in this study, we found changes in HDL-C to be associated with changes in Vo_2_peak and not with changes in body weight or fat mass. The association between ΔHDL-C and ΔVo_2_peak has previously been confirmed in older adults,^32^^,^^34^ and higher Vo_2_peak is associated with higher levels of HDL-C in men.^35^ This could indicate that in older adults, changes in fitness seem to be of more importance than changes in body composition for altering HDL-C levels. No difference was observed between the groups after 5 years; however, men in HIIT had a larger improvement in Vo_2_peak after 3 years compared with men in CON and MICT. As men in HIIT had a smaller reduction in HDL-C concentration after 5 years, this could indicate a delay in lipid metabolism in relation to fitness in older men.

### Strengths and Limitations

A main strength of this randomized controlled trial is the long intervention period and that the effect of exercise is examined in men and women separately. The use of both the intention-to-treat and the per protocol approaches has been requested by the literature.^12^ The Generation 100 study has previously used the intention-to-treat approach.^21^ In an unpublished study using the intention-to-treat approach, no changes were observed in HDL-C after 5 years of exercise. The per protocol approach allows us to examine the effect of exercising twice weekly for 5 years. However, a higher exercise volume could have altered our results as a higher exercise volume has previously been reported to be more important for altering blood lipids.^23^ The exercise programs are considered safe to perform by older adults as no severe events were reported during the 5 years.^21^ We performed sensitivity analysis whereby we excluded participants with CVD or taking an LMA. The results were similar to the main analysis, indicating that the presence of CVD did not affect our results. Men and women in HIIT had lower adherence compared with MICT at all follow-up times. Thus, HIIT might not be suitable for all older adults but seems to be efficient in older men who manage to regularly exercise with higher intensities. The adherence to exercise was based on data from questionnaires. A common weakness of self-reported questionnaires is response bias,^36^ and objective measures of physical activity have been recommended as a more accurate method.^37^ However, accelerometers’ activity thresholds are developed for younger adults and are inaccurate in measuring activities other than walking and running.^38^ The questionnaires used in this study have sensitivity in predicting physical activity level^39^ and thus are considered to be a suitable tool to assess exercise intensity. Diet was not part of the intervention; however, dietary data have found that participants did not report a change in their diet throughout the intervention period.^21^

## Conclusion

Our data found that HIIT seems to be the best strategy to prevent a decline in HDL-C during a 5-year period in men. No effect of exercise intensity was seen for older women. Changes in Vo_2_peak but not in body weight or fat mass correlate with changes in HDL-C in both men and women.

## References

[1] Liu H.H., Li J.J. (2015). Aging and dyslipidemia: a review of potential mechanisms. Ageing Res Rev.

[2] Bouaziz W., Malgoyre A., Schmitt E., Lang P.O., Vogel T., Kanagaratnam L. (2020). Effect of high-intensity interval training and continuous endurance training on peak oxygen uptake among seniors aged 65 or older: a meta-analysis of randomized controlled trials. Int J Clin Pract.

[3] Ugreninov E. (2005).

[4] Fernandez-Ballesteros R., Robine J.M., Walker A. (2013). Active aging: a global goal. Curr Gerontol Geriatr Res.

[5] (2019). 2019 ESC/EAS guidelines for the management of dyslipidaemias: lipid modification to reduce cardiovascular risk [erratum appears in Atherosclerosis. 2020;292:160-162]. Atherosclerosis.

[6] Reseptregisteret 2011-2015 [The Norwegian Prescription Database 2011-2015]. https://www.fhi.no/globalassets/dokumenterfiler/rapporter/2015/reseptregisteret-2011-2015-norwegian-prescription-database-2-utg.pdf.

[7] Barter P. (2011). HDL-C: role as a risk modifier. Atherosclerosis Suppl.

[8] Rader D.J. (2003). High-density lipoproteins as an emerging therapeutic target for atherosclerosis. JAMA.

[9] Rubins H.B., Robins S.J., Collins D. (1999). Gemfibrozil for the secondary prevention of coronary heart disease in men with low levels of high-density lipoprotein cholesterol. Veterans Affairs High-Density Lipoprotein Cholesterol Intervention Trial Study Group. N Engl J Med.

[10] Kelley G.A., Kelley K.S., Tran Z.V. (2005). Exercise, lipids, and lipoproteins in older adults: a meta-analysis. Prev Cardiol.

[11] Wood G., Murrell A., van der Touw T., Smart N. (2019). HIIT is not superior to MICT in altering blood lipids: a systematic review and meta-analysis. BMJ Open Sport Exerc Med.

[12] Bouaziz W., Vogel T., Schmitt E., Kaltenbach G., Geny B., Lang P.O. (2017). Health benefits of aerobic training programs in adults aged 70 and over: a systematic review. Arch Gerontol Geriatr.

[13] King A.C., Haskell W.L., Young D.R., Oka R.K., Stefanick M.L. (1995). Long-term effects of varying intensities and formats of physical activity on participation rates, fitness, and lipoproteins in men and women aged 50 to 65 years. Circulation.

[14] Cobbold C. (2018). Battle of the sexes: which is better for you, high- or low-intensity exercise?. J Sport Health Sci.

[15] Stensvold D., Viken H., Rognmo O. (2015). A randomised controlled study of the long-term effects of exercise training on mortality in elderly people: study protocol for the Generation 100 study. BMJ Open.

[16] Gibbons R.J., Balady G.J., Beasley J.W. (1997). ACC/AHA Guidelines for Exercise Testing. A report of the American College of Cardiology/American Heart Association Task Force on Practice Guidelines (Committee on Exercise Testing). J Am Coll Cardiol.

[17] Berglund I.J., Soras S.E., Relling B.E. (2019). The relationship between maximum heart rate in a cardiorespiratory fitness test and in a maximum heart rate test. J Sci Med Sport.

[18] Borg G. (1970). Perceived exertion as an indicator of somatic stress. Scand J Rehabil Med.

[19] Helsedirektoratet [Norwegian Directorate of Health] (2011). Folkehelse: fysisk aktivitet: anbefalinger [Public health: physical activity: recommendations]. https://www.helsedirektoratet.no/tema/fysisk-aktivitet.

[20] Nes B.M., Janszky I., Aspenes S.T., Bertheussen G.F., Vatten L.J., Wisløff U. (2012). Exercise patterns and peak oxygen uptake in a healthy population: the HUNT study. Med Sci Sports Exerc.

[21] Stensvold D., Viken H., Steinshamn S.L. (2020). Effect of exercise training for five years on all cause mortality in older adults—the Generation 100 study: randomised controlled trial. BMJ.

[22] Katzmarzyk P.T., Leon A.S., Rankinen T. (2001). Changes in blood lipids consequent to aerobic exercise training related to changes in body fatness and aerobic fitness. Metabolism.

[23] Kraus W.E., Houmard J.A., Duscha B.D. (2002). Effects of the amount and intensity of exercise on plasma lipoproteins. N Engl J Med.

[24] Thompson P.D., Buchner D., Pina I.L. (2003). Exercise and physical activity in the prevention and treatment of atherosclerotic cardiovascular disease: a statement from the Council on Clinical Cardiology (Subcommittee on Exercise, Rehabilitation, and Prevention) and the Council on Nutrition, Physical Activity, and Metabolism (Subcommittee on Physical Activity). Circulation.

[25] Kodama S., Tanaka S., Saito K. (2007). Effect of aerobic exercise training on serum levels of high-density lipoprotein cholesterol: a meta-analysis. Arch Intern Med.

[26] Gordon B., Chen S., Durstine J.L. (2014). The effects of exercise training on the traditional lipid profile and beyond. Curr Sports Med Rep.

[27] Kessler H.S., Sisson S.B., Short K.R. (2012). The potential for high-intensity interval training to reduce cardiometabolic disease risk. Sports Med.

[28] Wilmore J.H. (2001). Dose-response: variation with age, sex, and health status. Med Sci Sports Exerc.

[29] Boukabous I., Marcotte-Chénard A., Amamou T. (2019). Low-volume high-intensity interval training (HIIT) versus moderate-intensity continuous training on body composition, cardiometabolic profile and physical capacity in older women. J Aging Phys Act.

[30] Hwang C.L., Yoo J.K., Kim H.K. (2016). Novel all-extremity high-intensity interval training improves aerobic fitness, cardiac function and insulin resistance in healthy older adults. Exp Gerontol.

[31] Maillard F., Rousset S., Pereira B. (2016). High-intensity interval training reduces abdominal fat mass in postmenopausal women with type 2 diabetes. Diabetes Metab.

[32] Kelley G.A., Kelley K.S., Tran Z.V. (2004). Aerobic exercise and lipids and lipoproteins in women: a meta-analysis of randomized controlled trials [erratum appears in *J Womens Health (Larchmt)*. 2005;14(2):198]. J Womens Health (Larchmt).

[33] Hill J.O., Thiel J., Heller P.A., Markon C., Fletcher G., DiGirolamo M. (1989). Differences in effects of aerobic exercise training on blood lipids in men and women. Am J Cardiol.

[34] Weltman A., Despres J.P., Clasey J.L. (2003). Impact of abdominal visceral fat, growth hormone, fitness, and insulin on lipids and lipoproteins in older adults. Metabolism.

[35] Park Y.M., Sui X., Liu J. (2015). The impact of cardiorespiratory fitness on age-related lipids and lipoproteins. J Am Coll Cardiol.

[36] Shephard R.J. (2003). Limits to the measurement of habitual physical activity by questionnaires. Br J Sports Med.

[37] Aamot I.L., Forbord S.H., Karlsen T., Støylen A. (2014). Does rating of perceived exertion result in target exercise intensity during interval training in cardiac rehabilitation? A study of the Borg scale versus a heart rate monitor. J Sci Med Sport.

[38] Sallis J.F. (2010). Measuring physical activity: practical approaches for program evaluation in Native American communities. J Public Health Manag Pract.

[39] Kurtze N., Rangul V., Hustvedt B.E., Flanders W.D. (2007). Reliability and validity of self-reported physical activity in the Nord-Trondelag Health Study (HUNT 2). Eur J Epidemiol.

